# Histogram analysis in the differentiation between adrenal adenomas and pheochromocytomas: the value of a single measurement

**DOI:** 10.1590/0100-3984.2022.0067

**Published:** 2023

**Authors:** Ana P. Teixeira, William Haddad Jr., Luan Oliveira Barreto, André Secaf, Livia M. Mermejo, Fabiano R. Lucchesi, Silvio Tucci Jr., Jorge Elias Junior, Carlos A. F. Molina, Valdair F. Muglia

**Affiliations:** 1 Faculdade de Medicina de Ribeirão Preto da Universidade de São Paulo (FMRP-USP), Ribeirão Preto, SP, Brazil; 2 Hospital de Amor, Barretos, SP, Brazil

**Keywords:** Adrenal glands, Adrenocortical adenoma, Adrenal gland neoplasms, Incidental findings, Pheochromocytoma, Tomography, X-ray computed, Glândulas suprarrenais, Adenoma adrenocortical, Neoplasias das glândulas suprarrenais, Achados incidentais, Feocromocitoma, Tomografia computadorizada

## Abstract

**Objective:**

To assess the diagnostic accuracy of histogram analysis on unenhanced computed tomography (CT) for differentiating between adrenal adenomas and pheochromocytomas (PCCs).

**Materials and Methods:**

We retrospectively identified patients with proven PCCs who had undergone CT examinations between January 2009 and July 2019 at one of two institutions. For each PCC, we selected one or two adenomas diagnosed within two weeks of the date of diagnosis of the PCC. For each lesion, two readers scored the size, determined the mean attenuation, and generated a voxel histogram. The 10th percentile (P10) was obtained from the conventional histogram analysis, as well as being calculated with the following formula: *P10 = mean attenuation – (1.282 × standard deviation).* The mean attenuation threshold, histogram analysis (observed) P10, and calculated P10 (calcP10) were compared in terms of their diagnostic accuracy.

**Results:**

We included 52 adenomas and 29 PCCs. The sensitivity, specificity, and accuracy of the mean attenuation threshold were 75.0%, 100.0%, and 82.5%, respectively, for reader 1, whereas they were 71.5%, 100.0%, and 81.5%, respectively, for reader 2. The sensitivity, specificity, and accuracy of the observed P10 and calcP10 were equal for both readers: 90.4%, 96.5%, and 92.6%, respectively, for reader 1; and 92.3%, 93.1%, and 92.6%, respectively, for reader 2. The increase in sensitivity was significant for both readers (*p* = 0.009 and *p* = 0.005, respectively).

**Conclusion:**

For differentiating between adenomas and PCCs, the histogram analysis (observed P10 and calcP10) appears to outperform the mean attenuation threshold as a diagnostic criterion.

## Abstract (pt)

**Objetivo:**

Avaliar a acurácia diagnóstica da análise por histograma na tomografia computadorizada (TC) sem contraste para a diferenciação entre adenomas adrenais e feocromocitomas (FCCs).

**Materiais e Métodos:**

Identificamos, retrospectivamente, pacientes com diagnóstico de FCC confirmado que foram submetidos a exames de TC entre janeiro de 2009 e julho de 2019 em duas instituições distintas. Para cada FCC, selecionamos um ou dois adenomas diagnosticados em até duas semanas da data do diagnóstico do FCC. Para cada lesão, dois leitores pontuaram o tamanho, determinaram a atenuação média e geraram um histograma com os *voxels* das imagens. O percentil 10 (P10) foi obtido a partir da análise convencional do histograma, além de ser calculado com a seguinte fórmula: *P10 = atenuação média -* (*1,282 × desvio-padrão*). O limiar de atenuação média, o P10 da análise por histograma (P10 observado) e o P10 calculado (P10calc) foram comparados em termos de acurácia diagnóstica.

**Resultados:**

Foram incluídos 52 adenomas e 29 FCCs. A sensibilidade, especificidade e acurácia do limiar de atenuação média foram de 75,0%, 100,0% e 82,5% para o leitor 1, respectivamente, e de 71,5%, 100,0% e 81,5% para o leitor 2, respectivamente. A sensibilidade, especificidade e acurácia do P10 observado e do P10calc foram idênticas para os dois leitores: 90,4%, 96,5% e 92,6%, respectivamente, para o leitor 1; e 92,3%, 93,1% e 92,6%, respectivamente, para o leitor 2. O aumento da sensibilidade foi significativo para ambos os leitores (*p* = 0,009 e *p* = 0,005, respectivamente).

**Conclusão:**

Para a diferenciação entre adenomas e FCCs, a análise por histograma (P10 observado ou P10calc) parece superar o limiar de atenuação média como critério diagnóstico.

## INTRODUCTION

Adrenal incidentalomas (AIs) are a common finding on cross-sectional abdominal imaging, with a prevalence ranging from 1.0% to 8.7%, depending on the age of the individual; that is, increasing with advancing age^(1,2)^. It is estimated that pheochromocytomas (PCCs) account for 3–7% of all AIs^(3)^. Conversely, although clinically silent PCCs were previously assumed to be uncommon, some recent studies have estimated that up to 30% of PCCs are diagnosed as an AI^(4,5)^. A PCC is usually diagnosed on the basis of typical symptoms, such as headache, tachycardia, facial flushing, and sweating^(4)^; elevated blood pressure; and elevated plasma levels of metanephrines—the O-methylated metabolites of catecholamines^(6,7)^. The knowledge that a substantial proportion of patients with a PCC are either asymptomatic or mildly symptomatic is concerning because these lesions are associated with an increased risk of severe cardiovascular complications related to the excessive production of catecholamines^(8,9)^. In this scenario, the search for reliable criteria to differentiate between adrenal adenomas (AAs) and PCCs when an AI is found has been the focus of numerous studies in the literature^(10)^. Some studies^(7,11)^, including a multi-institutional analysis^(11)^, have indicated that an AI with a mean attenuation ≤ 10 HU on unenhanced computed tomography (CT) scans may not require biochemical screening to exclude a PCC, although endocrinology societies continue to recommend such screening^(12)^. However, it is also well known that a significant proportion of AAs do not contain enough intracytoplasmic lipids and will therefore display a mean attenuation > 10 HU^(13)^; that is, they are the so-called lipid-poor adenomas (LPAs). In that scenario, the washout technique represents an alternative diagnostic criterion^(14,15)^. The technique compares the mean attenuation observed on delayed-phase images (acquired 10–15 minutes after contrast injection) with that observed in the venous and unenhanced phases. It is a reliable method to distinguish AAs from metastatic lesions^(16,17)^, with a few exceptions (e.g., hepatocellular carcinoma and renal cell carcinoma metastases). However, the method is inaccurate for distinguishing AAs from PCCs because the latter often present with marked washout^(18,19)^.

In recent decades, the analysis of voxels in a region of interest (ROI) placed on an AI (histogram analysis) has emerged as an alternative to improve the characterization of LPAs^(20,21,22,23)^. Histogram analysis suggests that any homogeneous adrenal lesion ≤ 4.0 cm with at least 10% negative voxels could be assumed to be an AA^(20)^. More recently, there has been renewed interest in histogram analysis because some studies have demonstrated that the 10th percentile (P10) can be calculated from the mean attenuation of the same single ROI on unenhanced CT images, as long as the voxel distribution is normal^(24,25)^. However, histogram analysis has been tested only for distinguishing adenomas, especially LPAs, from PCCs in a single study, conducted by Remer et al.^(26)^. In that study, the authors assessed the P10 by counting voxels. Accordingly, we conducted the present study to assess the diagnostic accuracy of histogram analysis, using either voxel counting or a single measurement, for differentiating between AAs and PCCs.

## MATERIALS AND METHODS

### Study population

This study was conducted at two separate centers and was approved by the institutional committees on human research of both centers. Due to the retrospective nature of the study, the requirement for written informed consent was waived. This work used the Radiology Information System and electronic pathology databases. Two radiologists (not involved with the imaging analysis) searched for “pheochromocytomas” in both databases and retrospectively identified the cases of all patients in whom a diagnosis of PCC was proven by biopsy or surgery and confirmed by histopathology, between January 2009 and July 2019. We selected one or two adenomas diagnosed within two weeks of the date of diagnosis of each PCC, prioritizing those diagnosed closest to that date, in order to avoid any chronologic bias. The two-week interval was chosen to ensure that patients were examined in the same CT scanner.

Among the 52 adenomas selected, the diagnosis was confirmed by histopathology (regardless of the size) in four, and the remaining 48 adenomas were diagnosed on the basis of the criteria established in the American College of Radiology (ACR) White Paper on incidental adrenal masses^(10)^: being homogeneous; measuring ≤ 4.0 cm; and remaining unchanged in size for at least 12 months. Of those 48 lesions, 27 (56.2%) showed signal loss on opposed-phase magnetic resonance imaging. The mean follow-up period for the patients with adenomas was 37.3 ± 23.1 months (range, 13–107 months).

**Figure 1:**
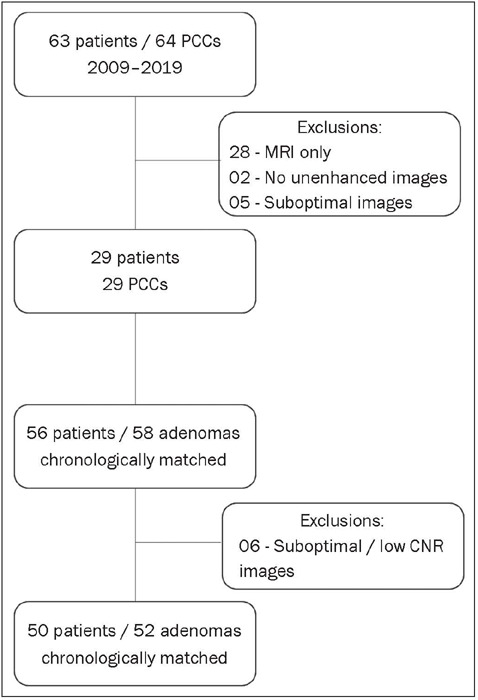
Flow chart showing the study selection process.

We applied the same exclusion criteria for AAs and PCCs: CT images unavailable; no unenhanced CT images available; suboptimal images (e.g., with extensive artifacts due to respiratory motion or metallic implants); low contrast-to-noise ratio (CNR) in the images; and thick (> 3.0 mm) slices. We retrieved the records for 64 PCCs, 35 of which were excluded: 28 because no CT images were available; two because the images were unenhanced; three because the images were suboptimal, including metallic artifacts in the adrenal area; and two because there was a low CNR due to obesity. Of the original 60 patients with AAs, six were excluded: four due to suboptimal images and two due to low CNR. Two patients had bilateral AAs. Therefore, we evaluated two groups: PCC (n = 29) and AA (n = 52). The flow chart in Figure 1 details the selection process.

### Clinical, biochemical, and demographic data

Two third-year radiology residents reviewed the electronic medical records of all enrolled patients. They retrieved (or confirmed) the following data related to each patient: age, sex, endocrinological test results, and histopathology findings when available.

### CT protocols and image quality

The CT examinations were conducted in one of three multidetector scanners: a Brilliance 16-slice scanner (Philips, Best, The Netherlands); a Somatom Sensation 64-slice scanner (Siemens, Erlangen, Germany); or a LightSpeed VCT 64-slice scanner (General Electric, Milwaukee, WI, USA). During image acquisition, the slice thickness was 1.0 mm or 1.25 mm, the tube voltage was 120 kVp, and the tube current was variable, as defined by patient size and body habitus^(27)^. All images were post-processed and reconstructed using a standard soft tissue algorithm at a collimation of 3.0 mm with no overlap reconstruction. When used, the pitch was set at 1:1 for all examinations.

Because of the retrospective nature of the study, the CT examinations were performed using different protocols according to the indication. However, we had access to data from unenhanced CT examinations (e.g., for urinary lithiasis) as well as from multiphasic CT examinations, including images acquired in the arterial, portal, and equilibrium phases, depending on the clinical indication, as well as in the delayed phase (e.g., during urological CT). The contrast-enhanced images were obtained in the arterial phase in accordance with the bolus tracking used in order to ensure synchronization. For the portal phase, the images were acquired at 60–70 s after the beginning of the injection. Iodinated contrast material—Visipaque 320 (General Electric) or Ultravist (Bayer Schering Pharma AG, Berlin, Germany)—was administered with a power injector, in a dose ranging from 100 mL to 120 mL (depending on the weight of the patient), at a rate of 2–4 mL/s.

The image quality was assessed by a senior radiologist, who placed ROIs ≥ 1.0 cm in diameter in the adrenal gland, liver, and spleen, as well as in the retroperitoneal fat adjacent to and in the adrenal gland lesion, to measure the CNR. A CNR > 2.0 was set as the threshold for considering an image to be of good quality^(28)^. The CNR was calculated using the following formula^(29)^:


CNR=(mean adrenal lesion density/SD of adrenal lesion density−mean paravertebral density)

### CT image analysis

Two independent readers with 4 and 5 years of experience in abdominal imaging, respectively, assessed the CT images. Both readers were blinded to the clinical, biochemical, and histopathology findings. The readers subjectively evaluated all adrenal lesions, assessing homogeneity on the unenhanced and contrast-enhanced images. They tested for the presence of calcifications and noted the size of the lesions.

Using the OsiriX Dicom Viewer (PixMeo, Geneva, Switzerland), the readers assessed unenhanced CT images and placed an ROI in each adrenal lesion at its largest diameter. From that ROI, they determined the mean and standard deviation of the attenuation. The information for that ROI was saved in XML format. On the basis of the mean attenuation and the corresponding standard deviation, they estimated the P10 according to the following formula^(30)^:


P10=mean−(1.28 standard deviation)

The value obtained with that formula was defined as the calculated P10 (calcP10).

Histogram analysis was carried out by a radiologist with more than 20 years of experience in abdominal imaging, who obtained the data from the saved XML file. The radiologist gathered the information obtained in the ROI to generate a Microsoft Excel file containing the attenuation values from each voxel, using those values to create a histogram of HU values. The same radiologist evaluated the histogram data and counted the number of voxels that were measured to determine the observed density value of the P10. The value obtained was designated the observed P10 of the histogram analysis (hereafter referred to as the observed P10).

For the estimation of diagnostic accuracy in this study, a lesion was assumed to be an AA when the following criteria were met: a mean attenuation < 10 HU on unenhanced images^(10)^; and an observed P10 or calcP10 < 0 HU. In clinical practice, two additional criteria can be combined with any other criterion from CT or magnetic resonance imaging^(10,31)^: the lesion should be homogeneous and should measure ≤ 4.0 cm. Here, we assessed diagnostic accuracy prior to applying those two criteria.

### Standard of reference

For the PCCs, the reference standards were the histopathological report and the biochemical test results. Surgical and histopathological confirmation was available for only four of the AAs. For the remaining 48 AAs, the reference standard was no variation in size during a follow-up period of at least one year, as well as measuring ≤ 4.0 cm and being homogeneous, in accordance with the previously mentioned recommendations of the ACR white paper on incidental adrenal masses^(10)^. Those criteria were verified by a third observer, a radiologist with more than 20 years of experience in abdominal imaging, before imaging analysis by the two readers.

### Statistical analysis

The Stata statistical software package, version 15 (StataCorp L P, College Station, TX, USA) was used for all analyses. The level of statistical significance was set at *p* > 0.05. The Shapiro-Wilk statistical test was employed to determine which continuous variables had a normal distribution including the histogram derived from the voxels. For normally distributed variables, Student’s t-test was used in order to compare the means between the two groups. The Mann-Whitney U test was employed to compare all other variables. The chi-square test was used in order to compare categorical variables. The diagnostic accuracies of the mean attenuation (using a cutoff value of 10 HU), the observed P10, and the calcP10 were compared by McNemar’s test. The interobserver agreement for density patterns (homogeneous or heterogeneous) and for the presence of calcifications was assessed with intraclass correlation coefficients via calculation of Cohen’s kappa (κ).

## RESULTS

The demographic data are shown in Table 1. The mean age of the patients was significantly lower in the PCC group than in the AA group (*p* = 0.001). There was no significant difference between the two groups for patient sex or lesion laterality. The measurements obtained by reader 1 indicated that 13 (25.0%) of the 52 AAs were LPAs (mean attenuation > 10 HU), whereas those obtained by reader 2 indicated that 16 (30.7%) were LPAs.

**Table 1:** Demographic and clinical data.

Variable	Adenomas (n = 52)	Pheochromocytomas (n = 29)	*P*
Age (years)*	60.0 ± 15.2 (9–87)	47.9 ± 16.7 (15–74)	0.001
Sex^†^			
Female	36 (69.2)	20 (69.0)	0.98
Male	16 (30.8)	9 (31.0)	
Laterality^†^			
Right	25 (48.1)	13 (44.8)	0.77
Left	27 (51.9)	16 (55.2)	
Reference standard^†^			
Follow-up	48 (92.3)	–	–
Surgery/pathology	4 (7.7)	29 (100.0)	

* Mean ± SD (range). ^†^ n (%)

When the imaging parameters on CT images were assessed (Table 2), the PCCs were found to be significantly larger than the AAs. On their longest axis, the PCCs were, on average, twice as large as the AAs (2.53 vs. 5.32 cm for reader 1 and 2.42 vs. 5.03 cm for reader 2; *p* < 0.0001 for both). Only three of the AAs (5.8%) were > 4.0 cm, whereas 12 of the PCCs (41.4%) were ≤ 4.0 cm. The mean attenuation value on unenhanced images was also significantly different between AAs and PCCs (4.84 vs. 36.58 HU for reader 1 and 5.83 vs. 36.63 HU for reader 2; *p* < 0.0001 for both). Similarly, the difference between AAs and PCCs for the observed P10 was also significant for both readers (*p* < 0.0001). The difference was also significant for the calcP10 (*p* = 0.01 for reader 1 and *p* = 0.001 for reader 2).

**Table 2:** Imaging parameters, by reader and by group.

Parameter	Reader	Adenomas (n = 52)	Pheochromocytomas (n = 29)	*P*
Size (cm), mean ± SD (range)	1	2.53 ± 1.43 (1.2 to 11)	5.32 ± 3.32 (1.4 to 16.0)	< 0.0001
	2	2.42 ± 1.44 (1.0 to 10.6)	5.03 ± 3.12 (1.3 to 14.9)	< 0.0001
Mean attenuation (HU), mean ± SD (range)	1	4.84 ± 15.44 (–27 to 44)	36.58 ± 8.31 (11 to 47)	< 0.0001
	2	5.83 ± 14.81 (–29 to 49)	36.63 ± 8.27 (12 to 49)	< 0.0001
calcP10 (HU), mean ± SD (range)	1	–23.04 ± 16.67 (–54 to 27)	14.55 ± 10.32 (–14 to 34)	< 0.0001
	2	–24.83 ± 17.06 (–64 to 34)	13.32 ± 10.25 (–19 to 30)	< 0.0001
Observed P10 (HU), mean ± SD (range)	1	–23.11 ± 16.51 (–51 to 28)	14.58 ± 10.4 (–15 to 35)	0.01
	2	–24.96 ± 17.0 (–59 to 35)	13.31 ± 10.61 (–20 to 31)	0.001
Calcifications, n (%)	1	1 (1.9)	5 (17.2)	0.01
	2	1 (1.9)	4 (13.8)	0.03
Homogeneity, n (%)	1			
	Homogeneous	43 (82.7)	7 (24.1)	< 0.0001
	Heterogeneous	9 (17.3)	22 (75.9)	
	2			
	Homogeneous	42 (80.8)	7 (24.1)	< 0.0001
	Heterogeneous	10 (19.2)	22 (75.9)	
Normality, n (%)	1	45 (86.5)	26 (89.7)	0.67
	2	45 (86.5)	24 (82.8)	0.65

**Figure 2:**
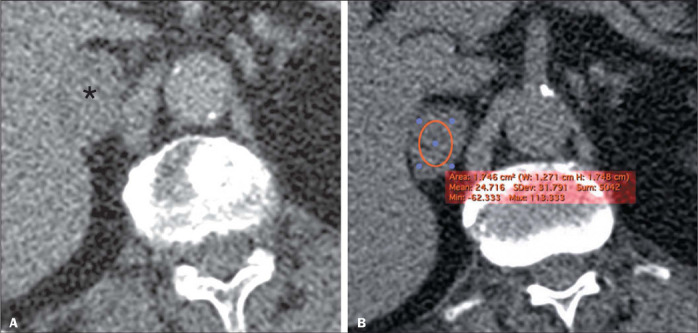
A 53-year-old female patient. A: Axial contrast-enhanced CT scan, in the venous phase, showing a right-sided homogeneous adrenal lesion measuring 2.8 cm (asterisk). B: Axial unenhanced CT scan at the same level with an ROI drawn at the center of the lesion, showing a mean attenuation of 24.7 HU, which is suggestive of an LPA (although not meeting the criteria at this point). However, the histogram analysis P10 and calcP10 showed that there was more than 10% negative voxels, further suggesting a diagnosis of LPA. At this writing, the patient is asymptomatic and the lesion has been stable for 62 months.

The AAs were homogeneous in 43 (82.7%) cases for reader 1 and in 42 (80.8%) for reader 2 (Figure 2). For both readers, the PCCs were homogeneous in only seven (24.1%) of the 29 cases. The difference in this distribution was statistically significant (*p* < 0.0001 for both readers). The interobserver agreement for homogeneity was excellent (κ = 0.87; *p* = 0.00001). Among the AAs, both readers identified calcifications in just one lesion (1.9%). Among the PCCs, readers 1 and 2 identified calcifications in five lesions (17.2%) and four lesions (13.8%), respectively. The difference between the two groups in the frequency of calcifications was statistically significant (*p* = 0.01 for reader 1 and *p* = 0.03 for reader 2). The interobserver agreement for calcification was also excellent (κ = 0.82; *p* = 0.00001).

**Figure 3:**
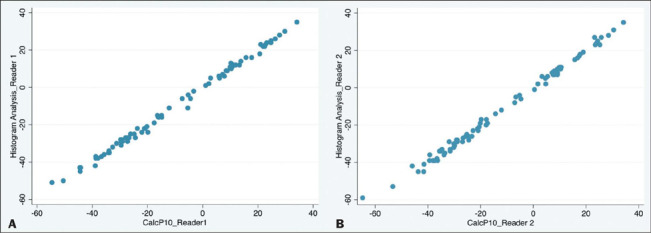
Scatter plot showing a strong positive correlation for the P10 observed on a histogram analysis (y-axis) and that calculated from a single ROI on an unenhanced CT scan (x-axis), by reader 1 (A) and reader 2 (B).

The distribution of the mean attenuation values of all voxels was Gaussian in 87.6% of the patients for reader 1 and in 85.1% for reader 2. There was no significant difference in that proportion between the AA and PCC groups: 86.5% of AAs for both readers; and 89.7% and 82.8% of PCCs for readers 1 and 2, respectively. This high frequency of Gaussian distribution is relevant because it is a requirement for using the formula to calculate the P10 via the mean and standard deviation. The correlation between the observed P10 and the calcP10 was strong for both readers (Figure 3): r = 0.9981 (*p* < 0.0001) for reader 1 and r = 0.9975 (*p* < 0.001) for reader 2.

**Figure 4:**
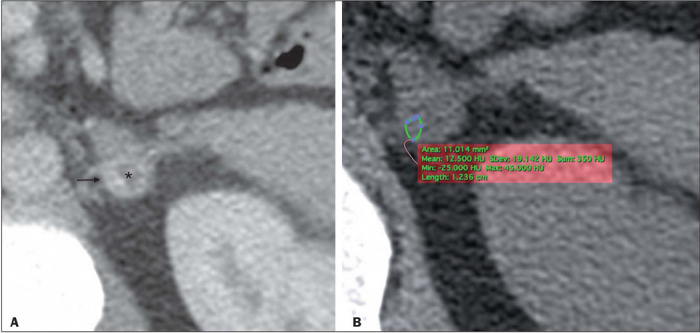
A 40-year-old male patient with symptoms and laboratory test results indicative of a PCC. A: Contrast-enhanced CT scan, in the arterial phase, showing a hypervascular 1.5-cm lesion on the left adrenal gland (arrow) with a central cystic area (asterisk). B: Axial unenhanced CT scan at the same level with an ROI drawn to avoid the cystic portion. The mean attenuation was 12.5 HU. The histogram analysis P10 and calcP10 showed more than 10% negative voxels. Aside from the clinical and biochemical setting, this could be assumed to be an AA, based on histogram analysis and calcP10 criteria, were it not for the heterogeneity of the lesion.

When assessing the diagnostic criteria for the mean attenuation criterion, observed P10, and calcP10 (Table 3), we observed that the sensitivity and accuracy for diagnosing AAs were higher for the observed P10 and calcP10 than for the mean attenuation criterion. For reader 1, the sensitivity of the 10 HU mean attenuation cutoff value was 75.0% (95% CI: 61.0–85.9), whereas it was 90.4% (95% CI: 79.0–96.8) for the observed P10 and the calcP10 (*p* = 0.009). For reader 2, those values were 71.1% (95% CI: 52.9–86.9) and 92.3% (95% CI: 81.5–97.9), respectively (*p* = 0.0005). For both readers, the specificity of the mean attenuation criterion was 100% (95% CI: 88.0–100.0). Among the 29 PCCs evaluated, readers 1 and 2 found that one (3.4%) and two (6.9%), respectively, showed more than 10% negative voxels. The specificity of the observed P10 and calcP10 was the same for reader 1—96.5% (95% CI: 82.2–99.9)—because one small (1.5-cm) PCC had a cystic portion (Figure 4). Outside of that cystic area, the lesion showed more than 10% negative voxels. For reader 2, the specificity of the observed P10 and calcP10 was also the same—93.1% (95% CI: 77.2–99.1). In addition to the small PCC mentioned above, a large (5.4-cm) PCC containing a large cystic area showed 15.4% negative voxels. The higher diagnostic accuracy was significant for reader 1 and for reader 2 (*p* = 0.05 and *p* = 0.03, respectively). In the AA group, 18 (34.6%) of the 52 lesions were LPAs (mean attenuation > 10 HU) and all of them were correctly assigned by the observed P10 and calcP10, indicating that a subgroup analysis would show higher sensitivity. The lower specificity was not significant for reader 1 (*p* = 0.09), although it was for reader 2 (*p* = 0.02). It is noteworthy that the two PCCs showing more than 10% negative voxels were heterogeneous with cystic areas and that the larger one was > 4.0 cm.

**Table 3:** Diagnostic accuracy of the mean attenuation criterion, observed P10, and calcP10.

	Reader 1					Reader 2				
Method	Sensitivity	Specificity	PPV	NPV	Accuracy	Sensitivity	Specificity	PPV	NPV	Accuracy
Mean attenuation criterion										
%	75.0	100.0	100.0	63.2	82.5	71.1	100.0	100.0	65.9	81.5
95% CI	(61.0–85.9)	(88.0–100.0)	—	(51.7–73.3)	(72.5–90.0)	(52.9–86.9)	(88.0–100.0)	—	(54.8–74.8)	(71.3–89.2)
Observed P10										
%	90.4	96.5	97.9	84.8	92.6	92.3	93.1	96.0	87.1	92.6
95% CI	(79.0–96.8)	(82.2–99.9)	(87.2–99.9)	(70.8–92.8)	(84.6–97.2)	(81.5–97.9)	(77.2–99.1)	(86.3–98.9)	(72.4–94.6)	(84.6–97.2)
CalcP10										
%	90.4	96.5	97.9	84.8	92.6	92.3	93.1	96.0	87.1	92.6
95% CI	(79.0–96.8)	(82.2–99.9)	(87.2–99.9)	(70.8–92.8)	(84.6–97.2)	(81.5–97.9)	(77.2–99.1)	(86.3–98.9)	(72.4–94.6)	(84.6–97.3)

PPV, positive predictive value; NPV, negative predictive value.

## DISCUSSION

Our data indicate that histogram analysis, using either voxel counting or the P10 formula, can help differentiate between an LPA and a PCC on unenhanced images without sacrificing specificity. This criterion, if confirmed in future studies, could be a powerful adjuvant technique for assessing AIs on CT images.

In 1998, Korobkin et al.^(14)^ and Szolar and Kammerhuber^(15)^ reported on the use of washout to characterize LPAs. That criterion was initially assumed to be effective for the differentiation between PCCs and AAs. However, Happel and Heinz-Peer^(19)^ subsequently showed that PCCs could have washouts in the same range as those of AAs. That claim was confirmed in other, similar studies^(32,33)^. Patel et al.^(34)^ showed that 33% of PCCs had either absolute or relative washout values meeting the cutoff criteria for AAs, as well as that half of those PCCs were homogeneous in the four phases studied: unenhanced, arterial, venous, and equilibrium. Of greater relevance in that study, all of the PCCs had a mean attenuation on unenhanced images above the 10 HU cutoff.

Recently, Canu et al.^(3)^ and Sane et al.^(7)^ reported that risk of being a PCC is negligible for homogeneous lesions measuring ≤ 4.0 cm and showing a mean attenuation ≤ 10 HU; international guidelines then recommended that no further endocrinological screening be performed for lipid-rich adenomas^(11,12)^. These studies were important because a previous study, from the early 2000s, reported that some adrenal lesions with a mean attenuation ≤ 10 HU were found to be PCCs^(35)^. Our findings are in keeping with those of the more recent studies: none of the PCCs in our sample had a mean attenuation ≤ 10 HU.

With the emergence of voxel counting, histogram analysis has been established using the rule of 10% negative voxels for characterizing an adenoma^(20)^. Several studies have confirmed the increased diagnostic accuracy when compared to the mean attenuation on unenhanced images^(21,22,23,24)^. Hsu et al.^(24)^ and Rocha et al.^(25)^ recently introduced a simpler, faster method of applying the criterion of 10% negative voxels, without voxel counting, based on a statistical formula to estimate the P10 of any Gaussian distribution. To date, only Remer et al.^(26)^ assessed the P10 criterion for distinguishing AAs from PCCs. However, those authors compared an AA group to a non-AA group that included metastases and PCCs, without reporting an isolated comparison between AAs and PCCs. They reported that the sensitivity of histogram analysis was not superior to that of the mean attenuation, the former being 69.5% and 72.4%, respectively, for two readers. They also found no significant difference between the mean attenuation and the P10 criterion in terms of the overall specificity, and they did not report the specificity for PCCs alone.

Of the 29 PCCs evaluated in the present study, one (3.4%) and two (6.9%) were found to show more than 10% negative voxels by readers 1 and 2, respectively. Therefore, the specificity of observed P10 and calcP10 was 96.0% for reader 1 and 93.1% for reader 2. However, in our sample, in contrast to what was found by Remer et al.^(26)^, the sensitivity of the observed P10 and calcP10 was found to be significantly greater than that of the mean attenuation criterion, for both readers (*p* = 0.009 and *p* = 0.0005 for readers 1 and 2, respectively), as was the overall accuracy of the observed P10 and calcP10. In the two cases of PCCs with more than 10% negative voxels, both had cystic areas. One possible explanation for this increased proportion of negative voxels is that there could be microscopic cystic areas that were not perceived when the ROIs were positioned. In both cases, the marked heterogeneity of the lesions would have prevented a presumptive diagnosis of AA. In our sample, the PCCs were significantly larger than the AAs, which could have been due to the fact that biopsy/surgical confirmation was an inclusion criterion, which unquestionably created a selection bias. Larger PCCs tend to be heterogeneous and are more likely to be symptomatic, therefore being more likely to be biopsied or surgically resected.

Another important parameter when assessing AIs is size. There is a consensus that surgical resection is indicated for adrenal masses measuring > 4.0 cm (excepting cysts and myelolipomas), regardless of any imaging criteria or functional status in the endocrinological evaluation^(36)^. In our study, 41.4% of the PCCs measured ≤ 4.0 cm on their longest axis. Only one of those PCCs had more than 10% negative voxels. That lesion was heterogeneous, with a well-defined cystic portion (Figure 4).

Our study has some limitations. First, it was a retrospective study, with an increased risk of biases, especially selection bias, as cited for the PCCs. We aimed to minimize the risk by using appropriate inclusion and exclusion criteria. Second, the sample was small, which reflects the relatively low incidence of PCCs. Although AAs are common lesions, we included a small portion of them (at a ratio of 2:1) to avoid inducing significant discrepancy between the two groups, which could be up to 50 times, considering the high prevalence of AAs. Third, for the group of AAs, we used predominantly follow-up, instead of histopathological analysis, as the reference standard. However, surgical approaches (or even biopsy) are not indicated in major international guidelines for small, nonfunctioning adrenal lesions. The great majority of AAs should simply be monitored for confirmation. In addition, we applied the same criteria mentioned by experts in the ACR White Paper^(10)^, and those criteria have been used in several previous studies^(15,16,17,18,20,21,22,23)^.

In conclusion, our data indicate that histogram analysis using the estimation of the P10 of voxels from a single measurement can be used for differentiating between nonfunctioning AAs and PCCs. This is important because it increases the value of histogram analysis for the characterization of LPAs.
